# Supplementary figures and images for: Differential modularity of the mammalian Engrailed 1 enhancer network directs sweat gland development
Source: PLoS Genet. 2023 Feb 6;19(2):e1010614. doi: 10.1371/journal.pgen.1010614 (PMC9934363; doi:10.1371/journal.pgen.1010614)

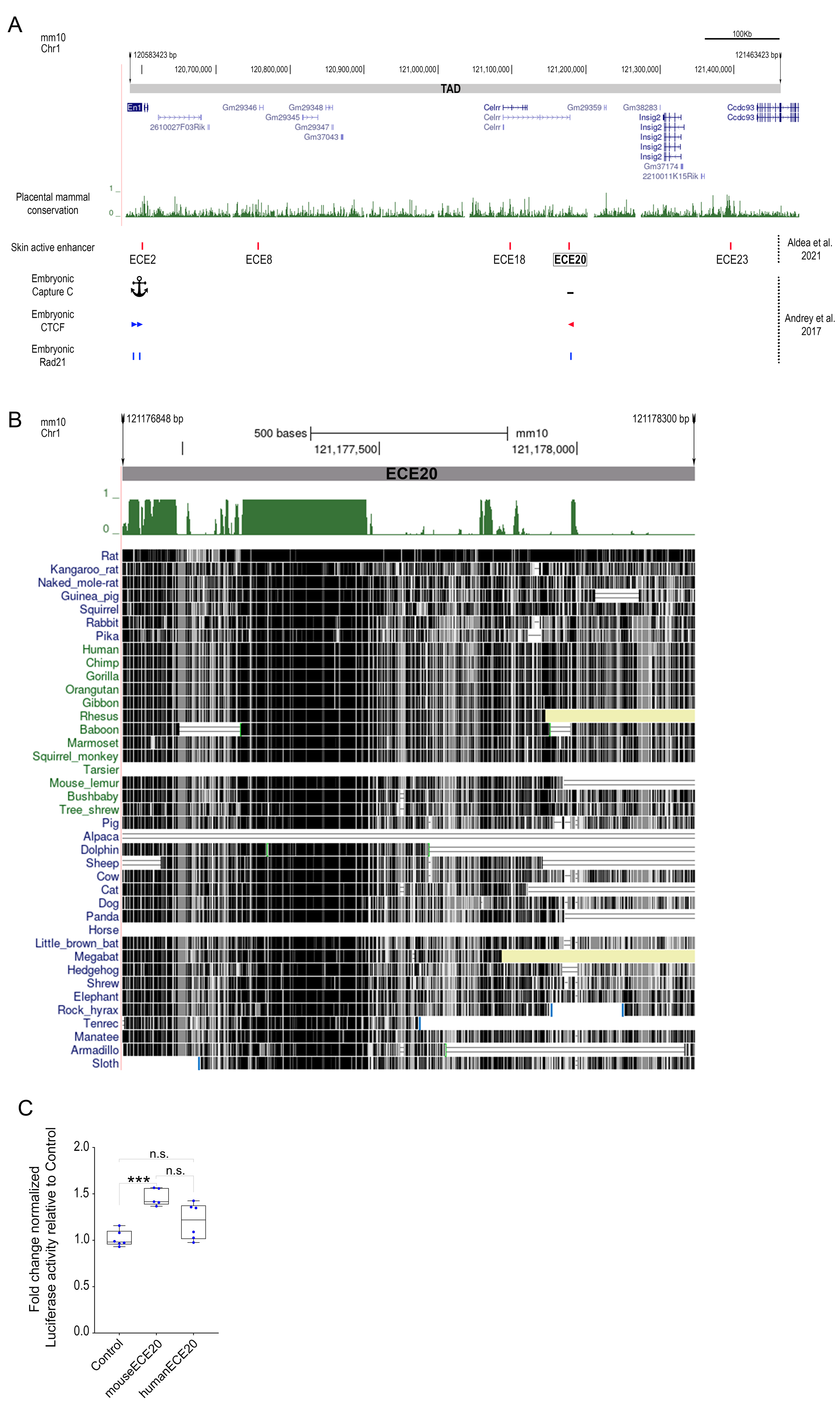

Supplement: S1 Fig — (A) Relative genomic position of Engrailed 1 candidate enhancer (ECE) 20 (boxed) and features of the ECE20-containing topologically associated domain (TAD, solid gray rectangle). Genomic positions of additional positive ECEs (red vertical lines) previously reported in Aldea et al. 2021 [24]. PhastCons scores based on alignment of placental mammals are depicted in green [46]. Called peaks from mouse embryonic limb and midbrain for interaction between the En1 promoter (anchor) and the genomic region containing ECE20 based on Capture-C (black, horizontal line), for CTCF enrichment (red and blue triangles show site location and directionality of CTCF sites), and for RAD21 enrichment (blue vertical lines) [33]. (B) Sequence alignment of mammalian genomes centered on ECE20 using mouse genome build mm10 as the base genome [46]. PhastCons scores for each position in the alignment are shown in green. Alignment and PhastCons scores are pulled from the USCS Genome browser (http://genome.ucsc.edu) [46] (C) Quantitative activity of mouse and human ECE20 orthologs in human GMA24F1A cultured keratinocytes, an immortalized human skin cell line that endogenously expressed EN1 [24,51,54]. The fold change in normalized luciferase activity relative to Control (empty vector) is plotted. Each dot represents a biological replicate. In (C) significance is assessed by one-way ANOVA and Tukey-adjusted P-values are reported. ***P<0.001, n.s. not significant. (TIF) [file pgen.1010614.s001.tif]

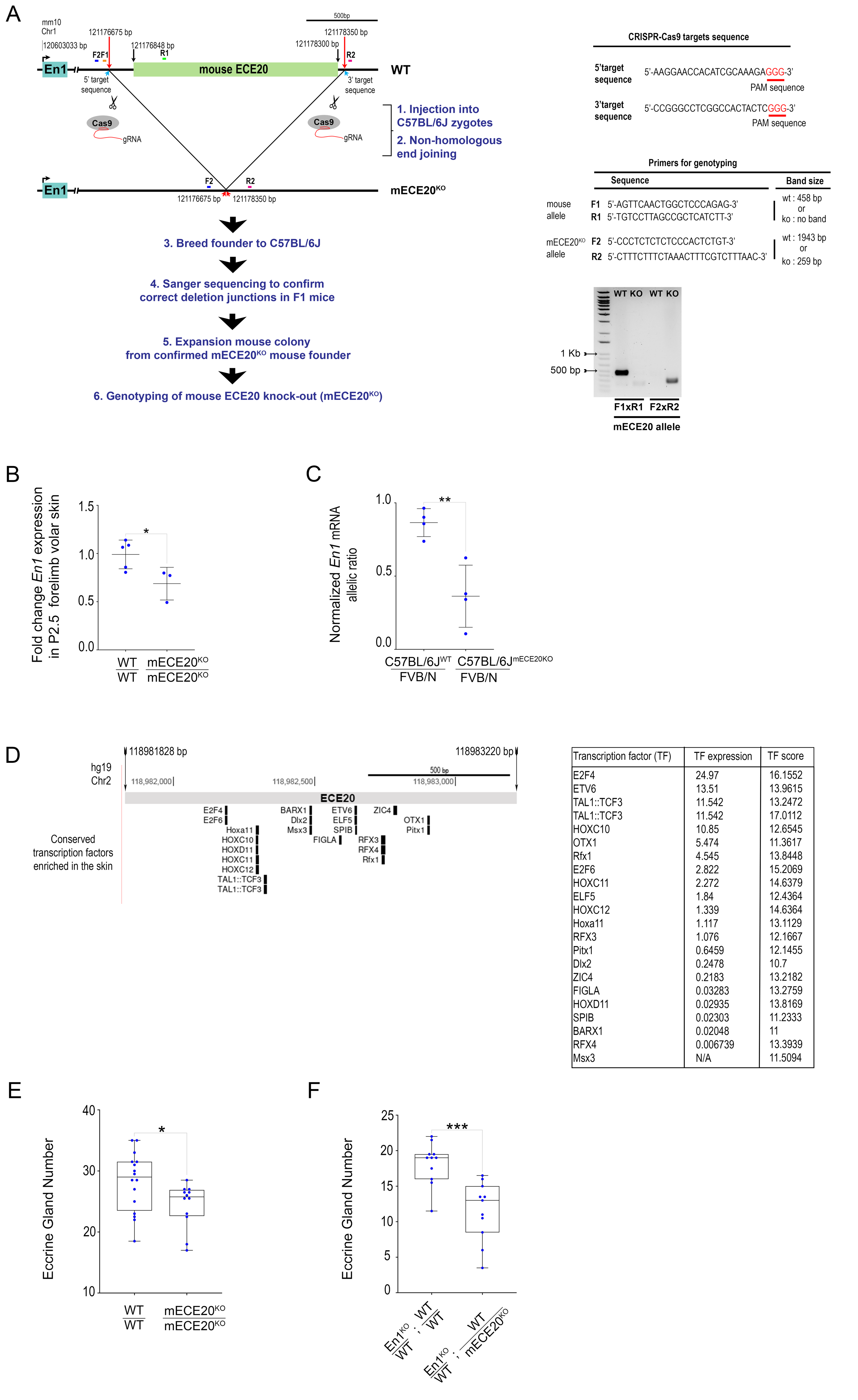

Supplement: S2 Fig — (A) Generation of an ECE20 knock-out mouse (mECE20KO) by CRISPR-Cas9 mediated genome editing. CRISPR-Cas9 target sequence and genotyping strategy are shown. Deletion junctions were confirmed by Sanger sequencing of F1 pups. (B) Fold change in En1 mRNA by qRT-PCR in P2.5 volar forelimb skin of wildtype (WT / WT), and mECE20KO homozygote (mECE20KO/ mECE20KO) mice relative to wildtype. (C) Normalized En1 mRNA allelic ratio in volar forelimb of wildtype at P2.5 of wildtype (C57BL/6JWT / FVB/N) and mECE20KO (C57BL/6J(mECE20KO) / FVB/N) hybrid mice. Ratios were normalized to the allelic ratio in F1 genomic DNA. Each point represents the mean value across three technical replicates of biological samples consisting of pooled P2.5 volar skins from both forelimbs of two or three mice. (D) Location and identity of in silico-predicted DNA binding motifs for transcription factors enriched in skin that are evolutionarily conserved between mouse and human ECE20. Relative expression of the cognate transcription factor (TF expression) and motif score (TF score) are shown. Motifs identified using funMotifs (tissue-specific functional motifs) [47]. (E) Quantification of interfootpad (IFP) eccrine gland number in adult volar forelimbs of WT / WT and mECE20KO/ mECE20KO mice. (F) Quantification of IFP eccrine glands in adult, volar forelimbs of En1 KO / WT; WT / WT and En1 KO / WT; WT / mECE20KO mice. In (B, C) dots represent an individual biological replicate. In (E, F) each point represents the average number of eccrine glands in the IFP across both forelimbs of a mouse. In (B, C) mean (line) with standard deviation are plotted. In (E, F) the median (line) and maximum and minimum are reported for each genotype. In (B, C, E, F) significance assessed by a two-tailed T-test. ***P<0.001, ** P<0.01, * P<0.05. (KO) knock-out. In (B, C) Rlp13a was used as housekeeping transcript for normalization. (TIF) [file pgen.1010614.s002.tif]

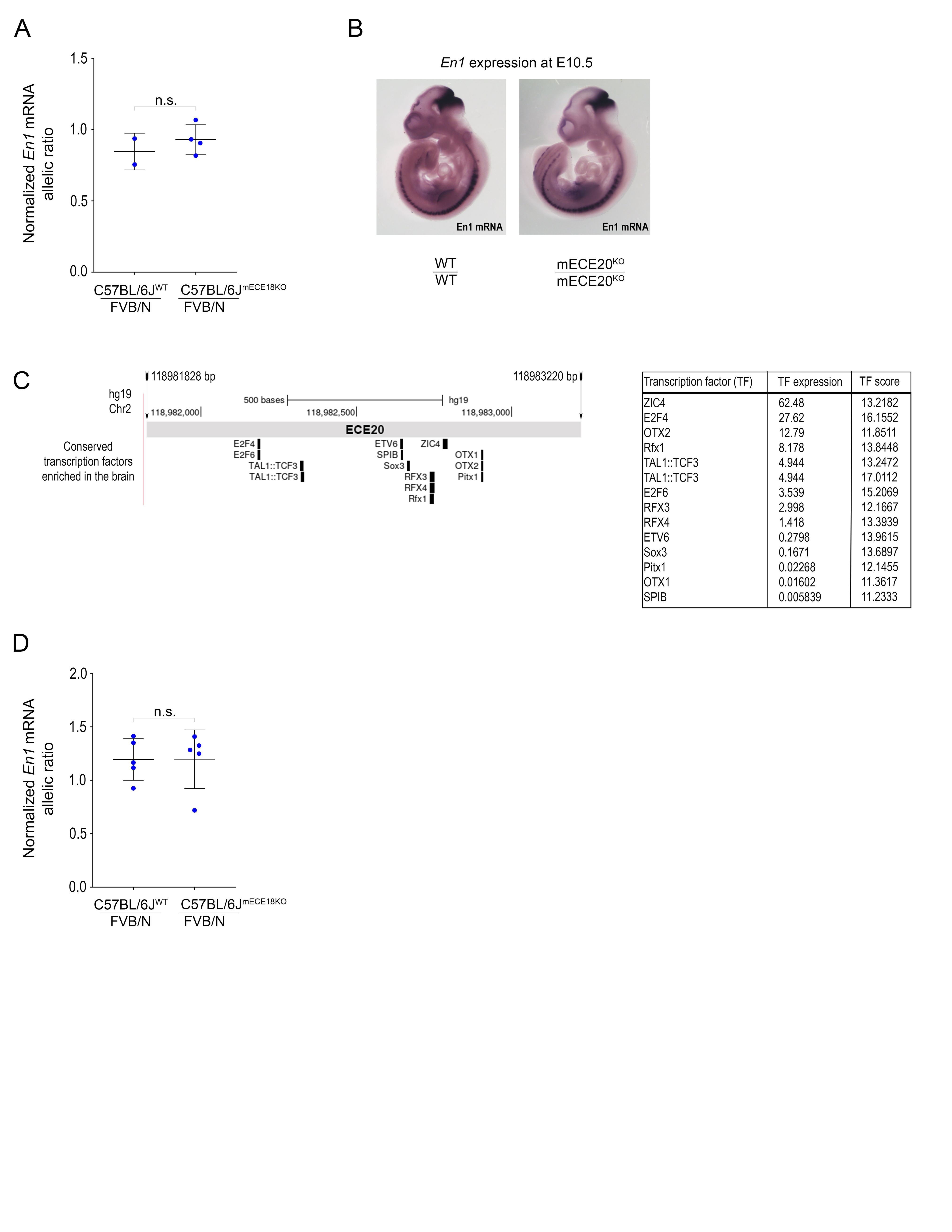

Supplement: S3 Fig — (A) Normalized En1 mRNA allelic ratios in forelimb autopods of wildtype (C57BL/6JWT / FVB/N) and mECE18KO (C57BL/6JmECE18KO / FVB/N) hybrid mice are plotted. (B) Whole-mount in situ hybridization for En1 in wildtype (WT / WT) and mECE20KO/ mECE20KO mice at E10.5. (C) Location and identity of in silico-predicted DNA binding motifs for transcription factors enriched in the brain that are evolutionarily conserved between mouse and human ECE20. Relative expression of the cognate transcription factor (TF expression) and motif score (TF score) are shown. Motifs identified using funMotifs (tissue-specific functional motifs) [47]. (C) Normalized En1 mRNA allelic ratios in midbrain-hindbrain of wildtype (C57BL/6JWT / FVB/N) and mECE18KO (C57BL/6JmECE18KO / FVB/N) hybrid mice. In (A, D) ratios are normalized to the allelic ratio in genomic DNA, and each point represents the mean value across three technical replicates of a pool of three or four mice for embryonic limb-bud in (A), or individual dissections of midbrain-hindbrain in (D) at E10.5. In (A, D) the mean (line) and the standard deviation are reported. In (A, D) significance assessed by a two-tailed T-test. n.s. not significant. (KO) knock-out. (TIF) [file pgen.1010614.s003.tif]

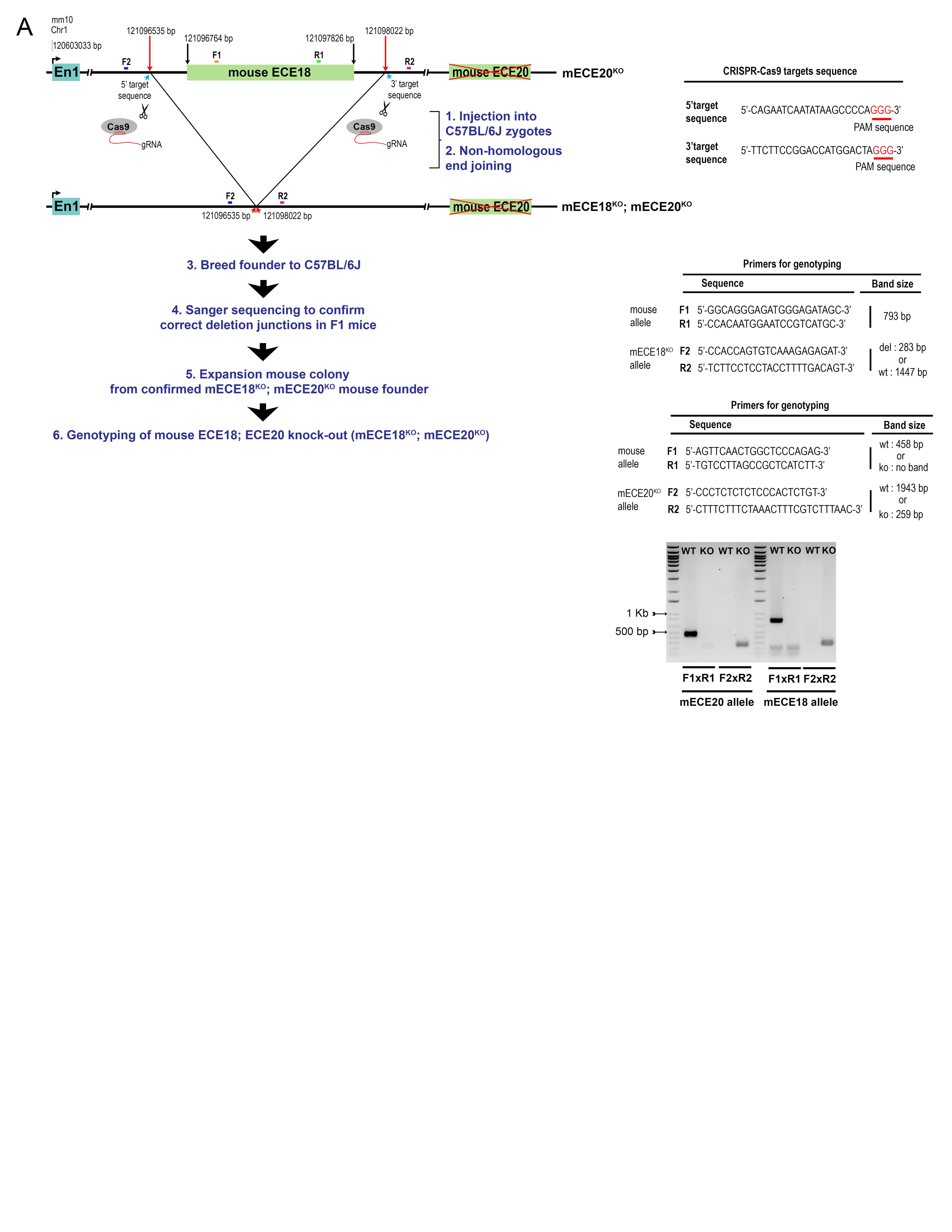

Supplement: S4 Fig — (A) Generation of an ECE18; ECE20 compound knock-out mouse (mECE18KO; mECE20KO) by CRISPR-Cas9 mediated genome editing. CRISPR-Cas9 target sequence and genotyping strategy are shown. Deletion junctions were confirmed by Sanger sequencing of F1 pups. (KO) knock-out. (TIF) [file pgen.1010614.s004.tif]
