# Supplementary material for: Differential modularity of the mammalian Engrailed 1 enhancer network directs sweat gland development
Source: PLoS Genet. 2023 Feb 6;19(2):e1010614. doi: 10.1371/journal.pgen.1010614 (PMC9934363; doi:10.1371/journal.pgen.1010614)
Supplement: S1 Table — (DOCX) [file pgen.1010614.s005.docx]

**Table S1:**

Primers used to subclone ECE20 orthologs in mouse transgenic assays^#^

| **ECE** | **Forward sequence** | **Reverse sequence** |
| --- | --- | --- |
| mouse-ECE20 | accaattgctcgaggCACATTCAAGGTCAATG | gtcaagcttccattatatagGCAGCAGTGAGTGTG |
| human-ECE20 | agtcgaccaattgctcgaggTACATCCAAGGCCAGTTTCTCC | cggtcaagcttccattatatagGCTACCGTGGGCGCCTGA |

^#^Lower case sequence indicates homology arms to Stagia3 vector

Primers used to subclone ECE20 orthologs into bidirectional luciferase reporter vector ^##^

| **ECE** | **Forward sequence** | **Reverse sequence** |
| --- | --- | --- |
| mouse-ECE20 | agagatttagaatgacaggcCACATTCAAGGTCAATGTCTCC | cttccattatatagaattCCGCAGCAGTGAGTGTGCGCGC |
| human-ECE20 | agagatttagaatgacaggcTACATCCAAGGCCAGTTTCTCCAA | aagcttccattatatagaattCCGCTACCGTGGGCGCCTGAGCAGAGCCG |

^##^Lowercase sequence indicates homology arm to vector

qRT-PCR primers used in this study

| **Name** | **Species** | **Forward sequence** | **Reverse sequence** |
| --- | --- | --- | --- |
| En1 | Mouse | GTGGTCAAGACTGACTCACAGC | GCTTGTCTTCCTTCTCGTTCTT |
| Fgf8 | Mouse | CATGGCAGAAGACGGAGAC | CATGCAGATGTAGAGACCTGTC |
| Fgf18 | Mouse | ACGTGGATGCGGAAGTC | CCTGCACTTGCCTGTGTT |
| Pax5 | Mouse | TCTGGAAGTCCCTACAGCCACC | GGTCAGTGACGGTCATAGGCG |
| Rpl13a | Mouse | CAGTGCGCCAGAAAATGC | GAAGGCATCAACATTTCTGGAA |

Primers used in allelic discrimination assay

| **Name** | **Species** | **Forward sequence** | **Reverse sequence** |
| --- | --- | --- | --- |
| En1 | Mouse | GAGCAGCTGCAGAGACTCAA | CTCGCTCTCGTCTTTGTCCT |
