# Supplementary material for: Differential modularity of the mammalian Engrailed 1 enhancer network directs sweat gland development
Source: PLoS Genet. 2023 Feb 6;19(2):e1010614. doi: 10.1371/journal.pgen.1010614 (PMC9934363; doi:10.1371/journal.pgen.1010614)
Supplement: Attachment — Submitted filename: RESPONSE TO REVIEWERS.docx [file pgen.1010614.s008.docx]

**RESPONSE TO REVIEWERS:**

**“Differential modularity of the mammalian Engrailed 1 enhancer network directs sweat gland development”**

We are grateful for the thoughtful feedback the reviewers provided and have addressed all requested changes/clarifications in the revised manuscript. A point-by-point response to reviewer comments is provided below and all requested changes are referenced here and highlighted in yellow in the revised manuscript file.

**Reviewer comments are in bold text, responses are in** plain text**.**

**Reviewer #1 :**

**1. The authors find that ECE20 deletion affects En1 levels in volar skin and in the midbrain-hindbrain region. In volar skin, this causes reduced numbers of eccrine sweat glands. Do the authors also observe abnormalities in the brain? These are predicted from their argument in the Discussion that ECE20 mutation would be detrimental to brain function and therefore selected against.**

We are pleased to provide the reviewer with additional data, now incorporated into the manuscript, that demonstrate the impact of ECE20 disruption on the embryonic brain. Consistent with the ECE20 enhancer controlling the magnitude but not the spatial expression pattern of *En1* in the midbrain-hindbrain, we have analyzed *En1* expression in the E10.5 brain by *in situ* hybridization and find that this is unchanged between WT and ECE20KOs (Figure S3B, see also lines 245 – 248 in the revised main manuscript). Taking this into account, we used qRT-PCR to quantify the expression of *Pax5*, *Fgf8*, and *Fgf18*, which are in a feedback loop with *En1* and mediate patterning the midbrain-hindbrain. We find that expression of all three transcripts is significantly downregulated in the midbrain-hindbrain region of ECE20KOs as compared to sibling controls (Figure 3E, see also lines 248 - 250 in the revised main manuscript). Thus, while we do not observe gross morphological changes in the brains of ECE20KOs at E10.5, these expression data demonstrate that ECE20 is required for the maintenance of a key neural circuit. While a full characterization of the brain of ECE20 mutants is currently underway in our laboratory and beyond the scope of the submitted study, these additional data are consistent with the proposed model that mutations in ECE20 would have implications for broader neural phenotypes. In addition to the new data in the revised results, we also allude to this point in the discussion (lines 338 -341 in the revised main manuscript).

**2. Do the authors observe predicted transcription motifs in ECE20 that are conserved between mouse and human, as would be expected given the apparently similar activity of ECE20 in the two species? Do these motifs differ from those seen in ECE18?**

We are pleased to provide these data. We scanned the ECE20 sequence looking for predicted, evolutionary conserved (human/mouse) DNA binding motifs for transcription factors previously reported to be enriched in skin. We report the results of this analysis in Figure S2D (lines 208 – 210 in the revised main manuscript) also provided below (Response Figure 1A). We additionally performed the same *in silico* scan on ECE18 (Response Figure 1B). While we find some overlap in predicted sites, and a number of differences, we note that prediction algorithms alone are prone to yield false positives. Thus, while we agree that such data are interesting to include, we are hesitant to speculate on the biological significance of such overlap or differences in the context of this manuscript without biochemical validation of binding to the cognate transcription factor in the appropriate developmental context. Studies beyond the scope of this manuscript are needed to determine the mechanistic basis for ECE18 and ECE20 activities.

**Response Figure 1.** Evolutionary conserved, predicted DNA binding motifs for transcription factors enriched in the skin. Relative gene expression (TF score) and motif score (TF score) are shown. Motif *in silico* analysis generated by funMotifs (tissue-specific functional motifs).

**3. Along these lines, are there any transcription factors/transcription factor families predicted to bind conserved motifs in ECE20 that are expressed in the embryonic midbrain as well as in volar skin?**

Similar to the analysis described in Response 2 above, we scanned the ECE20 sequence looking for predicted, evolutionary conserved motifs (human/mouse) for transcription factors with high expression in the brain. We found that DNA motifs such as Tal1 ::TCF3, E2F4, Rfx1, Rfx3, and Zic4 are expressed and shared between both skin and brain tissues. We now include the results of this analysis in Figure S3C (lines 248-250 in the revised main manuscript), see also Response Figure 2A below). We also refer to differences in transcription factor binding as a possible explanation for the difference between ECE20 activity in the skin/brain vs the embryonic limb bud in the revised discussion (lines 324 – 327 Revised manuscript). However, for the reasons stated above, we refrain from extensive discussion of the significance of these findings in the absence of functional validation. Current studies in our laboratory are exploring the biological significance of these sites but are beyond the scope of the presented study.

**Response Figure 2A.** Predicted, evolutionary conserved DNA motifs in ECE20 for transcription factors with enriched expression in the skin and brain, respectively. Relative gene expression (TF expression) and motif score (TF score) are shown. Motif *in silico* analysis was generated by funMotifs (tissue-specific functional motifs).

**4. The legend for supplemental Figure S1 requires editing for the English.**

Revised as requested.

**Reviewer #2:
1. Was High-C performed independently on the mouse dorsal limb and brain domains at P2.5 and P10.5? It is possible that these two regions temporal as well as regional differences in TAD configurations that might account for the differential response. Please clarify.**

Hi-C was not performed in our study. We cite Capture-C data from mouse embryonic limb buds and brains reported in Andrey et al. Genome Res (2017). To clarify this point, we have amended Figure S1A to include the reference to Andrey et al (2017) directly within the figure body. In addition, we have amended the relevant Results section to further highlight that we used these previously published data as evidence for contact between ECE20 and the *En1* promoter (lines 226-227 in the revised main manuscript text) .

The reviewer raises an interesting point given the proximity of *En1* to the TAD boundary and the evidence that loci at such positions can be more prone to incorporation into neighboring domains due to TAD variability (Luppino et al. 2020). To address the reviewer’s question with respect to the etiology of context-specific ECE20 activity, we have therefore amended the Discussion to highlight this point and state that additional studies are needed to determine whether the differential activity of ECE20 in the limb bud and the brain/skin can be attributed to variability in local chromatin configuration and proximity of En1 to the TAD boundary or to context-dependent availability of the transcription factors that regulate the different En1 enhancers (lines 324 – 327 in the revised main manuscript text).

**2. The viral enhancer-reporter constructs test human and mouse ECE20 function without regard to the TAD configuration. While it shows that human and mouse ECE20 are capable of regulating gene expression in the limb and skin, it doesn’t necessarily mean that they perform this function in their native state. Please discuss.**

We agree that transgenic assays do not equate with endogenous function. As described in the manuscript, we have evaluated the endogenous function of mouse ECE20 by deleting this enhancer from the mouse genome to generate the ECE20 knock-out (ECE20KO) and analyzing *En1* expression and eccrine phenotypes in this mutant as compared to wildtype siblings. We report that ECE20 knock-out mice have reduced ectodermal *En1* expression, that this effect is in *cis*, and that eccrine gland number is reduced in these animals, demonstrating that endogenous ECE20 is necessary to upregulate *En1* to promote eccrine gland formation in the mouse (Figures 2-4 of the revised manuscript).

We agree with the reviewer that the transgenic reporter assay for the human homolog in the human patch assay demonstrates that the endogenous human ECE20 enhancer may be similarly active *in vivo*  but does not conclusively prove this point. Importantly, this is the gold standard assay available to test the functionality of a human enhancer that functions in the context of developing human skin. This is because there are currently no *in vitro* systems that recapitulate human eccrine gland development, and the ECE20 enhancer does not function in cultured human or mouse keratinocytes (Figure S1C), which are amenable to Crispr-I and would allow us to address endogenous human ECE20 function. We raised these points in the Results (lines 163 -167; lines 181 -183 in the revised main manuscript text) and Discussion section (lines 295 -298 in the revised main manuscript text). That said, the high degree of sequence conservation between ECE20 human and mouse homologs (a point of contrast with the primate specific ECE18 enhancer which shows accelerated divergence between humans and all other species), and the comparable activity of the two homologs in the respective human and mouse transgenic assays, we believe provide compelling evidence for a conserved endogenous functionality of the human ECE20 homolog (lines 295 -298 of the revised main manuscript text).

**Minor
• ECE should be defined in abstract**

We have added the definition of ECE to the abstract as requested (Line 29 in the revised manuscript).
